# Overview of somatization through three historical lenses and future directions

**DOI:** 10.1007/s44192-026-00398-4

**Published:** 2026-02-18

**Authors:** Vidula Garde, Meryl Churchill, Jaimi Greenslade, Kerrianne Watt, Andrew J. Mallett

**Affiliations:** 1 Townsville Hospital and Health Service, Townsville, QLD Australia; 2 College of Public Health, Medical and Veterinary Science, James Cook University, Townsville, QLD Australia; 3 College of Medicine and Dentistry, James Cook University, Townsville, QLD Australia; 4 College of Healthcare Sciences, James Cook University, Townsville, QLD Australia; 5 Emergency and Trauma Centre, Royal Brisbane and Women’s Hospital, Brisbane, QLD Australia; 6 IMB and Faculty of Medicine, The University of Queensland, Brisbane, QLD Australia; 7 Department of Psychology, Townsville Hospital and Health Service, 100 Angus Smith Drive, Douglas, QLD 4814 Australia

## Abstract

The term ‘Somatization’ refers to the mind-body interactions that manifest as physical symptoms without an identifiable medical cause. Here, we aim to trace the conceptual developments in this area through three historical lenses with a view to understanding the evolution of the concept and its impacts on current healthcare provision. We note that despite considerable changes regarding the concept of somatization and related disorders, there is still a gap in understanding and, therefore, conceptualising of these disorders as they present across healthcare settings. We examine the key developments in this area and suggest future directions based on contemporary understanding and the gaps thereof. Based on the above evidence, we suggest an alternative framework, both from research and clinical perspectives, proposing a new direction, extending the understanding of these disorders to emergency care settings.

## Introduction

Somatization is generally defined as the experiencing and communicating of psychological distress as physical symptoms or conditions for which a physical aetiology cannot be readily demonstrated, and seeking medical help. Somatisation-related disorders, therefore, represent unique exemplifications of mind-body interactions that are challenging to conceptualise. The understanding of this concept is rooted in the concept of Hysteria *(definitions to follow)*—a term which itself has a long and convoluted history. For modern medicine, firstly, the term ‘somatization’, initially used to denote Freudian conceptualisation of (conversion) Hysteria [1], has since been used to denote Briquet’s conceptualisation of Hysteria. Secondly, since somatization presents as a phenomenologically diverse range of functional symptoms and symptom clusters that can be mistaken for physical disorders, it is classified under different diagnostic entities across various treating specialties (such as non-cardiac chest pain in Cardiology and nonspecific abdominal pain in Gastroenterology) [2]. Thirdly, a lack of definitional clarity, ambiguous uses of the term somatization, and the use of various terms to denote the construct of somatization, are some of the clinician and researcher challenges in perusing related literature.

In order to bring clarity to the construct, we *aim* to critically trace the historical development of the archetypal concept of Somatization, (through varying definitions), from historical perspectives, consider current challenges, and propose future directions, particularly its applications across healthcare settings.

### Defining somatization

The term “somatization” originates from a mis-translation of a German term “*organsprache*” (“organ language”) —first used by Stekel (1943), who defined it as a bodily disorder that arises as the expression of “*deep-seated neurosis*” [1].

Somatization has been defined as “*an idiom of distress in which patients with psychosocial and emotional problems articulate their distress primarily through physical symptomatology.*” [2]. Bridges and Goldberg [3] define *Somatizers* using the following criteria:

- Consulting behaviour: a patient seeks medical help for somatic manifestations of psychiatric illness without presenting with psychological symptoms,
- Attribution to physical rather than psychiatric disease,
- The psychiatric disorder is detectable by standardised research criteria, and.
- Treatment of psychiatric disorder results in symptom alleviation.

Lipowski’s definition, which is one of the most frequently used, defines somatization [4, 5]. as “*a tendency to experience and communicate psychologic distress (or psychosocial stress) in the form of physical symptoms*” and “*seek medical help*”.

All conceptualizations note that physical attributions are made by persons somatising, despite the absence of demonstrable organic cause and repeated reassurance to the contrary. Synthesising the above definitions, we can understand the term somatization to denote *the experiencing of physical symptoms*,* for which no organic cause can be found despite appropriate investigations. It is associated with psychological distress and behaviourally characterised by frequent help-seeking.*

As defined above, somatization refers to a *process* rather than a particular disorder. Several disorders are said to exemplify this archetypal process. We use the term ‘somatization-related disorders’ to refer to those clinical conditions where this archetypal process is said to underlie the manifestation of physical/somatic symptoms. These may be taken to include historical classifications such as *Hysteria*, or relatively more recent terms used to denote these manifestations, such as *Functional Somatic Syndromes*,* Somatoform Disorder*,* Somatization Disorders*,* Somatic Symptom Disorder*,* Medically Unexplained Symptoms*,* Persistent Physical Symptoms* and the more recent *Bodily Distress Disorder.* This conceptual overlap between the above entities has resulted in some theorists noting the indistinct boundaries and commonalities between these syndromes [6–9]. sometimes referring to them collectively as ‘somatizing disorders’ [10]. The concept has evolved over time, with each subsequent iteration of diagnostic systems, such as DSM, revisiting the concept of somatization to reflect advances in scientific understanding.

However, despite some conceptual overlap, the terms do not refer to the same clinical entity (Table 1).

**Table 1:** Conceptual overlap and demarcation between various somatization-related terms and diagnostic entities

Term	Description	Definition, scope and areas of overlap	Classificatory system
Somatization	Process	Psychological distress expressed as physical symptoms [4, 5]. It refers to a process that could be normative and is not a diagnostic entity. It is hypothesized to underlie SSD, BDD, FSS and MUS	Nil. (Although Somatization disorder was a DSM-III diagnostic category)
Functional somatic syndromes (FSS)	Descriptive category	Persistent physical symptoms without a clear organic cause (e.g., IBS, fibromyalgia) [11, 12]. Refers to a symptom or symptom cluster representative of a chronic condition related to a particular organ or functional system. It is often a specialty diagnosis and not a psychiatric one. It can overlap with SSD and BDD.	Nil
Medically unexplained symptoms (MUS)	Non-diagnostic label	Physical symptoms without a sufficient medical explanation [13–15]. It is a descriptive category.	Nil
Somatic symptom disorder	Clinical diagnosis	One or more distressing symptoms in conjunction with excessive thoughts/behaviour regarding them [16, 17]. The diagnostic entity focuses on the presence of distress and behaviour that is not entirely explainable by physical symptoms.	DSM-5
Bodily distress disorder	Clinical diagnosis	Multiple distressing symptoms in conjunction with symptom preoccupation and functional impairment [18, 19]	ICD-11

*Medically unexplained (physical) symptoms* (MUS or MUPS) refer to physical symptoms experienced by an individual for which no organic cause can be found [20]. When MUS/MUPS are associated with a single symptom or function, they are referred to as *Functional Somatic Syndromes* (FSS). Neither MUS nor FSS is a diagnostic category, although the latter is often used to denote MUS associated with a particular medical specialty and has specialty-specific names (e.g. non-specific abdominal pain). Most medical specialties have one or more FSS associated with them [20]. The term *Somatization*, however, refers to the experiencing of MUS as an expression of psychological distress.

The current review considers historical changes in the understanding of somatization-related disorders through three lenses—aetiological, phenomenological, and dimensional as subsequent evolutions of the concept of somatization. The review then discusses a major lacuna in diagnosing the more acute manifestations of the archetypal process, as seen in emergency care, and suggests possible future directions.

As per our conceptualisation, the developments in the field can broadly be summarised into three categories:

1. *Aetiological basis for conceptualisation*: these perspectives conceptualise somatization as a disorder of the mind, directly associated with maladaptive mental states or emotional processes.
2. *Phenomenological basis for conceptualisation*: these perspectives conceptualise somatization as a disorder with physical manifestations in the absence of an identifiable physical aetiology.
3. *Dimensional basis for conceptualisation*: these perspectives conceptualise somatization as a dimensional construct manifesting along a hypothetical continuum.

## Somatization as an aetiological construct

### Definition

These perspectives, which are some of the earliest conceptualisations in the field, relate the *expression of physical symptoms and the experience of psychological distress in a causal context*. In these conceptualisations, Hysteria, the wandering of the melancholy uterus, was held to be responsible for a number of unexplainable symptoms in women [21] (Hystera = uterus).

### Key developments

Relatively more modern aetiological theories, such as Psychoanalytical theories, generally conceptualise Hysteria as emerging from unconscious motivations or conflicts that are unable to be expressed for fear of repercussions. Causality is attributable to trauma or an internal psychological conflict, regarding unconscious (often sexual or aggressive) motivations.

In modern medicine, the concept owes its early development retrospectively to Sydenham [21–23], who, possibly acknowledging the Greek theorists, is said to have classified the disorder as *hysteria* in women and *hypochondriasis* in men, since men do not have a uterus [22, 23]. The concept of Hysteria has an important role in the genesis of psychoanalytical theory, with Charcot, Freud and Janet being some of its early exponents. Charcot was the initiator of the traumatic theory underlying hysteria, although his predominant emphasis was on sexual factors [24, 25]. Freud and Janet were both his students. Freud postulated that the underlying aetiology of Hysteria could best be explained through the process of *conversion* [26]. According to Freud, the process of ‘*conversion’* involves the separation of the affect associated with the incompatible idea, with exclusively sexual content. He believed that the conflict thus created was managed by being transformed into a somatic symptom [27, 28]. Freud’s focus on the subjective and symbolic meaning of symptoms made his approach unique for his time [29]. In Conversion models such as those postulated by Freud, the symptom serves to relieve the conflict (*primary gain)* or confer secondary or related benefits, e.g., reduction in everyday responsibilities *(secondary gain).* The concept of primary and secondary gain has remained largely unchanged since Freud [30]. 

Janet (1907) postulated that Hysteria was less a consequence of repression and conversion than a consequence of trauma-related dissociation at the point of memory formation. Janet maintained that it was not necessarily only sexual impulses that were aetiologically responsible for hysteria, but rather that symptom formation in hysteria was one of the ways in which trauma came to be expressed. His theory posited that symptom formation occurred through attentional narrowing and activation of memories that become dissociated from the main autobiographical memory base at the point of trauma- a process that he referred to as dissociation. Janet’s work has been postulated by theorists to have set the groundwork for understanding memory processes and recovery in trauma [31]. In light of the link between trauma and somatization [32], Janet’s construction of trauma-related processes has been postulated to be related to all forms of somatization [33]. 

Lipowski, whose definition is one of the most accepted definitions, was also influenced by analytical thought.

### Strengths and limitations

Theorists are divided regarding which of the earlier analytical theories of Somatization has been the more salient to contemporary practice [24, 25, 28, 34] despite their impact. The challenge with all analytical conceptualisations is that, whilst providing a unifying framework for the aetiology of the disorder, they do not provide objective criteria for diagnosis in contemporary medical settings. Concepts such as “conversion” or “hysteria” are also no longer relevant to current practice. Their greatest contribution is that they provide a theoretical framework for clinical formulation, and for clinicians to develop a deeper understanding of manifestations of psychological distress.

Regardless of the controversies in the field, it is because of these theorists that Somatization became a legitimate domain of medical study. The real contribution of aetiological theorists is that they have provided the groundwork for current research, future developments, and a holistic understanding. Early classificatory systems, such as DSM-I/II, were both influenced by the psychoanalytic school of thought.

### Implications for clinical practice

Early aetiological theorists have influenced both the contemporary classification and treatment of somatization-related conditions. The former is exemplified in the inclusion term ‘conversion disorder’ within the category of Functional Neurological Disorder of DSM-5; the latter is exemplified in trauma-informed psychotherapeutic approaches for those somatization-related conditions where trauma has a role to play. Additionally, the Freudian concept of primary and secondary gain and Janet’s concept of dissociation continue to influence case conceptualisations [35], thereby contributing to the treatment of these conditions in clinical practice.

## Somatization as a phenomenological construct

### Definition

The shift in focus from aetiology to phenomenology was precipitated by dissatisfaction with the subjective nature of the analytical construction of somatization-related disorders. The phenomenological perspective focuses on observable signs and symptoms that cannot be explained based on existing medical knowledge, despite appropriate medical investigation. The genesis of this perspective is attributed to Briquet’s work and his epidemiologic study of 430 patients with hysteria seen over 10 years [36, 37], and has informed the development of our understanding of somatization related disorders from DSM III onward.

### Key developments

Briquet defined Hysteria as a ‘*neurosis of the brain in which the observed phenomena consist chiefly of a perturbation of vital activities which serve as the manifestation of affective feelings and passions.’* Briquet considered the association of somatization-related presentations with psychological and social stressors, but he did not make any etiologic assumptions about them [36, 37]. Under the influence of Briquet’s perspective, Guze and others [38, 39], emphasised a need to shift in DSM-III from analytically influenced systems to phenomenologically-based, atheoretical, diagnostic classification. Consequently, and subsequently, somatization came to be conceptualised based upon clinical presentation of symptoms, for which no medical explanation was available at a point in time. These array of symptoms and syndromes came to be described as Medically Unexplained Symptoms (MUS) [40]. (A comprehensive review of historical developments in the field has been carried out by Fink [41]). Since it was Briquet’s concept of hysteria that laid the foundation for what we call somatization disorder in DSM-III and thereafter, DSM-III uses the term *Briquet’s syndrome* to describe somatization disorder. As Guze and others described *Briquet’s syndrome*, it referred to a poly-symptomatic disorder [39].

The 1980 task force on Nomenclature and Statistics of APA separated “Hysteria” from conversion, psychogenic pain and hypochondriacal disorders and called it *somatization disorder*. The revision involves a reduction in the number of symptoms and symptom clusters. DeSouza and Othmer [42] examined the utility and validity of a revised and less stringent construct for somatization and proposed the abridged criteria. Escobar and colleagues [43] proposed *Abridged Somatization* disorder for individuals in primary care with a lifetime prevalence of MUS [43].

Kirmayer and Robbins [44] propose an alternate conceptualisation separate from ICD and DSM systems and describe three forms of somatization particularly relevant to primary care. Their approach derives from the approach proposed by Bridges and Goldberg [3]:

i. Functional somatization—a characteristic of individuals with history of Medically Unexplained Symptoms,
ii. Hypochondriacal somatization—characterised by worry about having a serious illness despite the absence of evidence, and.
iii. Presenting somatization—somatic clinical presentations in patients with evidence of current major depression or anxiety disorder.

In operationalising the above conceptualisation of somatization in primary care, the three could be separated based on high levels of functional somatic distress, hypochondriasis, and exclusively somatic clinical presentations among patients with current major depression or anxiety. This approach was further refined by Craig et al. [45, 46]. The phenomenological approach, albeit with its several limitations [47] has been influential in the recognition of somatization-related disorders in primary care. (An excellent critique of this research has been provided by Gucht and Fischler [47]

### Strengths and limitations

Phenomenological perspectives on somatization have ensured a degree of objectivity in assessment and diagnosis. These perspectives have de-emphasised unconscious causation and focused on observable symptoms, thereby providing clinician guidelines for objective diagnosis. Moreover, the reduction of emphasis on the unconscious as aetiologically implicated in its causation meant that it could increasingly be diagnosed based on clinical signs and symptoms. The recognition that this was feasible, removed its diagnosis solely from the realm of specialist psychiatrists to the realm of general physicians and other specialists who could henceforth have access to the tools to diagnose this disorder in primary care. However, terms such as MUS increase the risk of iatrogenic harm due to investigations needed to rule out diagnoses before the condition can be labelled “medically unexplained,” and render the gap between the clinician and the patient more difficult to bridge [48–51]. It also results in potential stigmatisation of patients [48] as well as unnecessary investigations, adding to healthcare costs [52]. Furthermore, it is difficult to refer to somatization syndromes as being ‘medically unexplained’ given developments in the understanding of aetiological mechanisms which are common to various somatization-related disorders [53]. Additionally, the division of disorders according to the treating specialist results in fragmented conceptualisation, piecemeal understanding, and lack of comprehensive care [53]. This is particularly true for individuals who present with symptoms from multiple organ systems [53].

### Implications for clinical care

Although DSM-5 no longer uses the word “somatoform disorder” to describe the constellation of symptoms or symptom clusters which cannot be medically explained, and the term “medically unexplained” itself has come under criticism over the years; the lack of medical explanation for observable signs and symptoms continues to provide the clinician with a useful heuristic, which could alert them to the existence of somatization [54]. Somatization seen from the aetiological lens was firmly the domain of psychiatry. The phenomenological concept of functional somatic syndromes has opened somatization-related disorders to other medical specialties for identification and possible treatments.

## Somatization as a dimensional construct

### Definition

Dimensional conceptualisations of somatization, perceive somatization as a manifestation of an underlying dimension. Some theorists contend that ‘somatization’, ‘conversion’, and ‘dissociation’ are different manifestations of the same underlying pathology/pathological dimension [9, 55, 56]. These perspectives can be subsumed under the dimensional construct.

### Key developments

In general, somatization-related disorders have been conceptualised as lying on the following dimensions:

- *Somatic distress*: Both, DSM-5 and ICD-11 (as well as earlier versions of these classifications) acknowledge that somatization might lie on a continuum of symptom severity. Symptom severity could be a factor that determines where in the health-services continuum somatization-related disorders are seen, and the clinicians who see them [57–59]. Since they present more frequently to primary care than to psychiatric care [60, 61], it was argued that a specialist diagnostic system, such as DSM, misses the less severe end of the continuum [57]. For more effective identification and treatment, diagnostic criteria in primary care require positive diagnostic features rather than focusing on the exclusion of medical diagnosis alone [59]. Furthermore, merely the exclusion of medical diagnoses at a point in time does not imply that the presentation is necessarily for a functional condition without organic causation, nor can a disorder be defined in terms of the absence of other disorders.
- *Abnormal illness behaviours*: proposed by Pilowsky [62], using Mechanic’s [63] concept of Illness behaviour to postulate that all forms of somatization represent Abnormal Illness Behaviours. Pilowsky [62] defines Abnormal Illness Behaviour as the “*persistence of an inappropriate or maladaptive mode of perceiving*,* evaluating or acting in relation to one’s own state of health despite the fact that …. appropriate social agent has offered an accurate and reasonably lucid explanation of the nature of the illness and the appropriate course of management…”.* Other theorists, such as Ford, also agree that somatization can best be described as illness behaviours [6].

Kirmayer and Looper [64] suggest that we need to rethink the nomenclature and indeed conceptualisation of somatization based on further research and a better understanding of abnormal illness behaviours. Still others [65] maintain that abnormal illness behaviours could be influenced by underlying psychiatric conditions. There is a general agreement regarding the need for further research in the field [66].

### Strengths and limitations

Dimensional conceptualisations of somatization represent an acceptance that somatization is essentially a normal process and moves away from pathologizing every manifestation of this process. The challenge with all of the dimensional conceptualisations of somatization revolve around the fact that, in considering somatization as a normative dimension, it is often difficult to indicate the point at which a normative dimension becomes pathological [67].

### Implications for clinical care

More than other conceptualisations, it is the conceptualisation of somatization-related disorders as abnormal illness behaviours that has resulted in the shifting of focus from the symptoms to the behavioural response to the symptoms. This shifting of focus to abnormal illness behaviours has contributed significantly to the current DSM-5 and ICD-11 diagnoses of SSD and BDD, respectively.

## Recent developments

Recent approaches consider biopsychosocial processes in the development of somatic symptom-related disorders. This has included the shifting of attention from symptom manifestation per se to psychobiological factors such as central sensitisation [68], somatosensory amplification [69] and illness attribution [70]. These changes in perspective encompass the consideration of neurological differences such as alteration to brain networks involved in cognitive control, emotional regulation and processing, and stress and somatic-visceral perception [71, 72]. They reinforce the fact that there is evidence for abnormal central nervous processing in these syndromes [73]. In addition, newer theories also consider the predictive nature of the brain and the salience of nociceptive information. These theories are referred to as predictive processing conceptualisations [74]. Additionally these perspectives consider the role of stress and the contribution of allostatic load/overload to the emergence of somatization-related syndromes [75]. Consequently, both the ICD-11 and DSM-5 increasingly emphasise the behavioural aspects of presentation in conjunction with the physical symptom manifestation. These developments are indicative of a conceptual shift in understanding and classifying somatic symptom-related disorders.

At their inception, the concept of somatization and related disorders in their various iterations has evolved to best categorise a subset of individuals with specific symptoms, presenting in specialist settings. These conceptualisations are not necessarily applicable across all settings. In fact, the impetus for change in DSM-5 and ICD-11 comes from the fact that all conceptualisations of somatization-related disorders in ICD-10 and DSM-IV were criticised as being inadequate for primary care. Theorists such as Fink and others [76] note “*The present diagnostic systems reflect that they are based on studies in specialized settings and an adoption to primary care settings*,* where the patient may be much earlier in the course of illness*,* may be needed.*” It is in response to the need for timely diagnosis in primary care among other factors such as utility of the diagnosis for practicing clinicians in general [77] that some of the DSM − 5 and ICD − 11 revisions to the concept of somatization-related disorders were made. Additionally, it has also been argued that the previous diagnostic categories were said to be confusing due to the overlap of various diagnoses and the requirement for the symptoms to be medically unexplained [54]. The changes from DSM-III to DSM-IV and from DSM-IV to DSM-5 (in response to various challenges and controversies in classifying somatization and related disorders), comprise a successive reduction in the number of symptoms required to make a diagnosis. In continuation with these changes, DSM-5 [78], Somatic Symptom Disorder (SSD) criteria have the requirement for a single somatic symptom accompanied by excessive thoughts, feelings, or behaviours. It does not require that the symptom/s be medically unexplained. It does specify the duration (6 months) and states that the ‘somatic symptoms must be significantly distressing or disruptive to daily life’.

In a similar vein, major changes have also been introduced by ICD-11, where somatization-related disorders are primarily classified as Bodily Distress Disorder (BDD). The two main criteria for BDD involve the presence of bodily symptoms that are distressing to the individual and an excessive amount of attention paid to those symptoms. The concept that bodily distress per se, with attendant behavioural features, merits a diagnosis, is a significant departure from earlier classifications [79]. Interestingly, in response to the opinion that positive diagnostic features could be more effective than negative ones, both BDD and SSD do not require the absence of a medical condition but stipulate that, in those who have a (contributory) diagnosed medical condition, the symptom preoccupation needs to be more excessive than can be explained by symptom profile or severity. The chief difference in BDD and SSD is that Hypochondriasis (as illness anxiety disorder) has been retained within SSD in DSM-5, although it is classified along with obsessive-compulsive disorders (OCD) in ICD-11. Overall, these changes are seen as a modest improvement [18, 80], particularly in the conceptualising of “bodily distress in a non-dualistic manner” [79], whilst simultaneously raising concern regarding the impact of the lowering of the diagnostic threshold [81, 82]. The risks inherent in the lowering of diagnostic threshold involve overdiagnosis of somatization related disorders and an increase in attendant healthcare costs [52, 83, 84] as well as the risk of mislabelling physically ill individuals as having a mental disorder (since it is clinicians judgement which determines whether or not the anxiety and preoccupation regarding the somatic symptom is “excessive”). In the field trial, BDD has been found to have better diagnostic accuracy than ICD-10 somatoform disorder diagnosis [79]. On the other hand, BDD can be confused with similar-sounding diagnoses such as Bodily Stress Syndrome and Bodily Distress Syndrome [85], adding to diagnostic confusion [79]. Neither of the newer diagnostic classifications is without its critique, and these have been discussed extensively in research literature [54, 80, 86–88].

*In summary*, thus far, we have considered the development from the three lenses—aetiological focusing on causality; phenomenological focusing on manifestation; and dimensional focusing on the intrinsic property of symptom profile, which are simultaneously adaptive in normative form and maladaptive in their pathological form.

## Future directions

Viewing somatization-related disorders from a public health perspective it could also be of interest to see if the developments seen in the conceptual aspects of somatization-related disorders translate to service provision in a public health system.

**Fig. 1:**
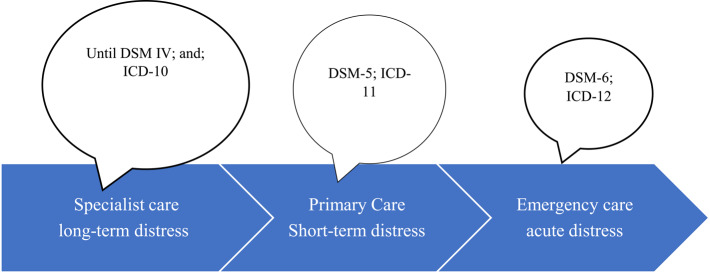
Healthcare service delivery as a continuum of temporality of somatic distress and development of diagnostic criteria for somatization-related disorders

We propose that *healthcare service delivery is a continuum of temporality of somatic distress*,* with acute distress presenting to ED*,* short-term distress to primary care*,* and long-term or chronic distress to specialist services*. Extending the logic of researchers such as Fink [76] we suggest that since diagnoses of somatization-related disorders have initially been attempted and solidified in their chronic manifestation (where they are seen by specialists), and have now been extended to short-term manifestation in primary care, it would be timely to consider the diagnostic implications in emergency care settings (Fig. 1). In the interest of providing comparable care over the continuum of manifestation, it is important that we extend the development of diagnostic criteria or algorithms to diagnosing somatization-related disorders in their most acute presentation, which is in ED.

Inasmuch as the controversies in the diagnosis of somatization-related disorders [6, 11, 89–93] can be considered to be attributable to the multifaceted nature of this disorder, and our incomplete understanding of mind-body interactions [94], they could also be attributable to varying clinical presentations and patient needs across healthcare settings. Just as it has been convincingly argued that primary care presentations for somatization-related disorders needed criteria that were more sensitive to primary care presentations, (as evidenced by the critique which necessitated and informed the current ICD-11and DSM-5 revisions), so too, it could also be argued, that somatization-related disorder presentations to other parts of the healthcare system, such as emergency care, have eluded diagnosis primarily due to lack of diagnostic sensitivity of the existing criteria to presentations in these settings.

In the latter context, an important future challenge for diagnostic refinement of somatization-related disorders could involve conducting epidemiological studies that examine the validity of SSD or BDD criteria across settings [95] and test theories that link diagnostic categories to aetiological processes or service needs [96]. Significant contributions in this regard are being made by the European Research Network to Improve Diagnosis, Treatment, and Health Care for Patients with Persistent Somatic Symptoms (EURONET-SOMA) group [53] and SOMACROSS (Persistent SOMAtic symptoms ACROSS diseases) [96] research unit, towards creating a scientific framework for systematic research regarding persistent physical symptoms across disease entities [96]. Similarly, the field might need to consider criteria for creating a consistent framework across healthcare settings, particularly in emergency care settings, where there are limited studies regarding somatization presentations.

It is acknowledged that there are several challenges in diagnosing somatization-related disorders in ED, including current definitions and conceptualisations [97]. The challenge in Emergency settings is that, frequently, the duration cannot be verified (both ICD-11 and DSM-5 stipulate a time period), frequent functional presentations in ED, such as functional cardiovascular symptoms [98], do not appear in the ICD 11 section on diseases of the circulatory system [79], have no reliable diagnostic features (duration cannot be reliably established, associated behavioural features such as high anxiety are normative in ED settings) and have no defined treatment pathway. However, there is evidence to suggest that somatization-related disorders present to ED across national borders [99], are responsive to health anxiety [100], and are common in frequent presenters [101]. The challenge of diagnosis is further compounded by the risk of missing a potentially life-threatening medical disorder, a dialectic requiring competent and delicate management, which, whilst it continues to challenge clinicians everywhere, has arguably more severe and immediate repercussions in ED than other healthcare settings [97]. Absence of ED-applicable diagnostic criteria can thus result in clinician dilemmas, regarding diagnostic accuracy and clinical risk, when faced with somatization-related disorders, which mimic high acuity, life-threatening conditions [97]. Somatization presentations in ED, therefore, contribute to high healthcare costs without necessarily improving patient outcomes. Since both economic-sustainability and patient-safety imperatives demand better criteria for diagnosing somatization-related disorders in ED, it is important to characterise and conceptualise somatization-related disorders in ED context.

Some theorists have sought to resolve the dilemma of diagnosis in ED by using special terms for MUS in ED and referring to it as EDMUS [99]. Burton and his colleagues from the EURONET- SOMA group [53] propose the creation of a single diagnostic entity, “*Functional* (for disorder of a function) *Somatic Disorder*” (FSD), occupying a “neutral categorical space” between physical and mental disorders. They suggest that this could be further sub-categorised depending on the number of systems affected, and additional specifiers used, depending on the relationship between the physical disease systems and/or presence or absence of psychological features. Whilst their approach makes no mention of ED, it could still be applicable in ED settings with appropriate specifiers for ED.

Combining Burton’s suggestion with the phenomenological approach of considering somatization-related disorder presentations (symptom constellations) unique to ED, might be one of the possible ways to conceptualise somatization-related disorders in ED.

Such criteria could consider both the presence of typical symptoms or symptom clusters that are not medically explainable in the ED contexts (e.g., low acuity presentations in high acuity settings) in conjunction with the presence of positive features of somatization, which could include repeated presentations and seeking of medical assurance as key behavioural features.

From the latter perspective, in the absence of ED-related diagnostic criteria for somatization diagnosis, a suggestion could be to possibly create a unique diagnostic entity or specifier for somatization-related disorder presentations in ED, which typically focuses on the phenomenology of frequent ED-related somatization presentations (such as atypical chest pain). In order to create a systematic understanding for conceptualising, identifying and diagnosing somatization-related conditions in ED we would like to suggest the following framework:

Firstly, using the clinical wisdom gained from the phenomenological approaches, the first step towards operationalising a definition of somatization-related disorders in ED could rely on epidemiological studies of frequent presentations in ED, which are not found to have physical causation based on current medical knowledge. Some earlier work by ED clinicians could be useful in this regard, such as the list of functional somatic syndromes in ED provided by Stephenson and Price [20].

Secondly, using the clinical wisdom gained from the etiological approaches and current DSM-5/ICD 11 guidance, ruling in the presence of current and active psychological distress symptoms, such as high anxiety or depression or behavioural correlates, including frequent low acuity presentations and observing abnormal illness behaviours as they pertain to typical ED presentations.

Thirdly, through studying the cross-sectional symptom profile of all presentations that meet these criteria.

Finally, through following the longitudinal course (2–5 years) of the presentations in ED with symptom profiles suggestive of somatization-related disorder.

Thus the steps involved in creating an ED specific diagnostic category could involve creating a provisional diagnosis or suggestive symptom profile based on earlier research, including aetiological and phenomenological approaches, applying these to the acute dimension of somatic presentations as seen in ED; carrying out epidemiological studies into these disorders with attendant psychological distress; considering individuals with a particular symptom profile suggestive of somatization manifestations in ED and carry out longitudinal studies.

Following the above suggestions could help us to develop diagnostic heuristics (and shared understanding) regarding the diagnostic criteria that could be appropriate for somatization-related disorders in ED. Once such symptom profile/s have been established, the research would need to focus on cross-cultural validity and sensitivity and specificity of the criteria, to manage the risk of overdiagnosis and overlap with existing diagnostic categories.

Appropriate diagnosis and management in ED could guide service provision through effective ED triage and referral pathways, be transformative in identifying and reducing fragmentation in care. Healthcare systems could then provide more comprehensive care, and ‘no man’s land’ would thus become ‘everyone’s territory’.

## Conclusion

Mind-body interactions resulting in physical symptoms without an identifiable medical cause are challenging to conceptualise, as exemplified through the various conceptualisations of somatization-related disorders. These disorders are frequently found in medical settings and are named after disorders with similar presentations. Both DSM-5 and ICD-11 have responded to the growing focus on primary care physicians’ need for separate diagnostic criteria for somatization-related disorders. Much, however, needs to be done for improving relevance to emergency care settings since there is a dearth of ED-appropriate criteria for diagnosing somatization-related disorders. Given that these disorders do present to the ED, it is desirable to explore cohesive and consistent diagnostic criteria applicable across settings or to consider creating a unique diagnostic category within ED contexts. This approach could involve using the wisdom gained from the three lenses and current biopsychosocial approaches to create a better understanding of somatization related disorders in emergency settings.

## Data Availability

No datasets were generated or analysed during the current study.
